# Studying the association between longitudinal mammographic density measurements and breast cancer risk: a joint modelling approach

**DOI:** 10.1186/s13058-023-01667-8

**Published:** 2023-06-09

**Authors:** Maya Illipse, Kamila Czene, Per Hall, Keith Humphreys

**Affiliations:** 1 Department of Medical Epidemiology and Biostatistics, Karolinska Institutet, Stockholm, Sweden; 2 Swedish eScience Research Centre (SeRC), Karolinska Institutet, Stockholm, Sweden; 3 Department of Oncology, Södersjukhuset, Stockholm, Sweden

**Keywords:** Breast cancer, Mammographic density trajectory, Joint model, Longitudinal study

## Abstract

**Background:**

Researchers have suggested that longitudinal trajectories of mammographic breast density (MD) can be used to understand changes in breast cancer (BC) risk over a woman’s lifetime. Some have suggested, based on biological arguments, that the cumulative trajectory of MD encapsulates the risk of BC across time. Others have tried to connect changes in MD to the risk of BC.

**Methods:**

To summarize the MD–BC association, we jointly model longitudinal trajectories of MD and time to diagnosis using data from a large ($N = 40{,}087$) mammography cohort of Swedish women aged 40–80 years. Five hundred eighteen women were diagnosed with BC during follow-up. We fitted three joint models (JMs) with different association structures; Cumulative, current value and slope, and current value association structures.

**Results:**

All models showed evidence of an association between MD trajectory and BC risk ($P < 0.001$ for current value of MD, $P < 0.001$ and $P =0.005$ for current value and slope of MD respectively, and $P < 0.001$ for cumulative value of MD). Models with cumulative association structure and with current value and slope association structure had better goodness of fit than a model based only on current value. The JM with current value and slope structure suggested that a decrease in MD may be associated with an increased (instantaneous) BC risk. It is possible that this is because of increased screening sensitivity rather than being related to biology.

**Conclusion:**

We argue that a JM with a cumulative association structure may be the most appropriate/biologically relevant model in this context.

## Introduction

A woman’s breast is a complex soft-tissue organ and its composition changes over time. Mammographic density (MD) reflects breast tissue composition; epithelial and stromal cells, collagen, and fat and varies extensively between individuals. MD is the most established image-based risk factor for BC [1–4]. MD also plays a major role in decreasing mammographic sensitivity [5–7], since both dense tissue and tumors appear white on mammograms.

Researchers have suggested that longitudinal trajectories of breast density can be used to understand changes in BC risk over a woman’s lifetime [8]. Several studies have tried to understand the changes of density with age using tumor free mammograms [9–12]. It has been shown that decline in MD with increasing age is most pronounced over the menopausal transition [13], and reaches a plateau around the age of 65 [9]. These trends are consistent across diverse groups of women around the globe, suggesting that they result from an intrinsic biological, most likely hormonal, mechanism [9, 10]. This pattern in density decline led researchers to draw parallels to Pike’s model for the rate of breast tissue ageing [14, 15]. This model suggests that the slowing rate of increase of age-specific BC incidence which is seen after menopause occurs because of a reduction in the rate of breast tissue ageing in postmenopausal women [16]. Boyd et al. [17] showed that age-specific cumulative MD is strongly associated with age-specific incidence of BC using cross-sectional data. They suggested that the cumulative MD profile is consistent with the accumulation of mutations with increasing time of exposure to MD, and that it may be considered as a tissue-specific marker of the biological processes underlying the rate of breast tissue ageing [9, 13]. No study in the literature has yet showed that cumulative value of MD at an individual level, i.e. using longitudinal data, is associated with BC risk.

Others studies have tried to connect changes in MD to the risk of BC via (association) analyses of longitudinal data. These have provided contrasting conclusions [8, 18–23]. These studies have used different methods for measuring MD, and have defined density change in different ways. They have used fairly standard statistical methods in their analyses, treating density measurements as fixed covariates. Recent studies [21, 22] have included density change as a time-varying covariate in a time-to-event analysis (Cox model). This approach is not ideal. Firstly, it assumes that the time-dependent covariate is exogenous, and secondly, it compensates for the fact that the covariate is not observed continuously by replacing unobserved values by the most recent observed value. This approach can provide biased parameter estimates, especially when there are irregular and infrequent measures of the marker. Moreover, it assumes that MD is measured without error. MD measurements are known to be noisy. Ignoring measurement error in a marker can lead to a severe underestimation of an association with a time-to-event/diagnosis process [24].

For observational studies with longitudinal data on MD and follow-up of cancer diagnoses it is appropriate to jointly model both processes, i.e. to fit a joint model (JM) [25]. In addition to accounting for the measurement error in the longitudinal outcome (MD), JM offers several options for modeling the association between the marker (MD) and the time-to-event (diagnosis) process [26]. JMs have been previously used to study the association between density/density change and death due to BC in BC patients [27]. As far as we are aware, there is only one publication [28] in which a JM has been used to study the association between MD and BC diagnosis/incidence. This work did not specifically model error in the measurements of density, and assumed a linear trajectory for the longitudinal marker and a simple model for the time-to-event process (current value association structure; see “Methods” section).

The objective of this paper is to explore the use of joint modelling of observational data, for investigating hypotheses that have been put forward to explain the association between the trajectory of MD and the risk of BC. We fit three JMs with different association structures to a large Swedish mammography screening cohort which has been previously used to study MD change and BC risk [22].

## Materials and methods

### Materials

KARMA is a population-based prospective screening cohort [29]. All women that participated in the national screening program at four mammography units in Sweden from January 2011 to March 2013, were invited to participate in the study. Women attending screening were enrolled, irrespective of how many previous screens they had attended. A total of 70,874 women were included. Informed consent was given for a continuous collection of mammograms. Using the Swedish personal identification number, KARMA has been linked to a number of registers, including the national quality register for BC. For the present study we excluded all women without informed consent and with missing information on MD, body mass index (BMI), hormone replacement therapy (MHT) and menopausal status at baseline (MP). We also excluded prevalent breast cancer cases, women with previous cancers, except non-melanoma skin cancer, women with any breast surgery, including those with breast enlargement and/or breast reduction. Moreover, we included only women aged between 40 and 74 at baseline, and ended follow-up at age 80. Because we investigate longitudinal MD, we started follow-up from the second screen after entrance to the cohort (most women - those older than the minimum screening age that had previously accepted screening invitations - had had several screens before entering the KARMA study). This selection (delayed entry) is handled appropriately in the statistical analysis (see “Methods” section). The final analysis included 40,087 women. Five hundred eighteen of these women were diagnosed with BC during follow-up. This number includes both in-situ and invasive cancers and cancers diagnosed both via screening and symptomatically. All participants signed an informed consent and the ethical review board at Karolinska Institutet approved the study.

Processed mammograms from the mediolateral oblique (MLO) view of both breasts were collected from full-field digital mammography systems. For women with BC, mammograms from the contralateral breast (i.e the breast which does not have a tumor) were used, whereas for women that were not diagnosed with BC before the end of follow-up, one side was selected randomly at baseline and follow-up measurements were based on mammograms from the same side. Dense area (cm$^2$) was measured using the STRATUS method which aligns mammograms from the same woman before taking density measurements [30]. We used area-based absolute density in our analysis, which is believed to be a more etiologically relevant phenotype of MD for BC risk than percent density, since it reflects the amount of tissue at risk of carcinogenesis [31].

### Methods

Joint modeling is an increasingly productive area of statistical research that has developed rapidly since the 1990s [25, 32–34]. JMs construct a mixed-effects model to describe the evolution of the longitudinal outcome (MD) over time, and simultaneously connect that process (as a time dependent covariate) to an outcome (BC) using a time-to-event model. JM accounts for loss of follow-up (correctly handles the potential association between the longitudinal measurements and drop-out) while taking random variation into account.

### Longitudinal submodel

For modeling MD over time we use a mixed-effects model [35]

1 $$\begin{aligned} y_i(t_{ij}) = m_i(t_{ij} )+ \epsilon _{ij},\ \epsilon _{ij} \sim N(0,\sigma ^2) \end{aligned}$$

where $y_i(t_{ij})$ is the *j*th observed longitudinal response of the continuous biomarker (MD) for the *i*th individual taken at time $t_{ij}$. Measurement error is incorporated through $\epsilon _{ij}$, and $m_i(t_{ij})$ is modeled as:

2 $$\begin{aligned} m_i(t_{ij}) = X_i^T (t_{ij}) \beta +Z_i^T (t_{ij}) b_i+u_i^T \delta ,\ b_i \sim N(0,D) \end{aligned}$$

where fixed effects $\beta$, with design matrix $X_i$ are shared across individuals (they represent the mean trajectory of the biomarker over time), and patient-specific random effects $b_i$ with design matrix $Z_i$ define the individual deviation relative to the mean trajectory. The random effects are assumed to have a multivariate normal distribution with mean zero and variance-covariance matrix *D*. It is possible to model nonlinear trajectories by including polynomials or splines of time in both $X_i$ and $Z_i$, and the effect of covariates on the trajectory can be modeled by including interactions with time in $X_i$ and $Z_i$. Baseline covariates are included using $u_i$ with regression coefficients $\delta$. The model naturally handles uneven spacing of repeated measurements.

### Time-to-event submodel

We use a proportional hazards model to model time to diagnosis. We assume that the hazard of experiencing the event of interest (cancer diagnosis) is dependent on a subject-specific characteristic of the longitudinal trajectory and that it can be formulated as

3 $$\begin{aligned} h_i(t) = h_0(t)\ \text {exp}(\gamma ^T w_i + f(m_i(t),b_i,\alpha )), \end{aligned}$$

where $h_0(t)$ describes the (baseline) hazard. The effect parameters $\gamma$ describe how the hazard varies as a function of explanatory covariates $w_i$. The parameter $\alpha$ quantifies the association between a priori selected features of the longitudinal process and the hazard for the event at time t. Several options for the function *f*() are possible and lead to different forms of the time-to-event submodel [26]. The first considered function is:

4 $$\begin{aligned} f(m_i(t),b_i,\alpha ) = \alpha \ m_i(t) \end{aligned}$$

This formulation assumes that the current ‘true’ level of MD is directly associated with the instantaneous risk of BC. The second considered function includes an additional term which is the current slope (rate of change) of MD:

5 $$\begin{aligned} f(m_i(t),b_i,\alpha ) = \alpha _1 m_i(t)+ \alpha _2 m_i'(t) \end{aligned}$$

The coefficients $\alpha _1$ and $\alpha _2$ express the strength of the association between the current value and rate of change of the (true) subject trajectory (of MD) at time *t* and the instantaneous risk of BC diagnosis. The last considered function is based on the cumulative value of MD:

6 $$\begin{aligned} f(m_i(t),b_i,\alpha ) =\alpha \int _{t_0}^t\! m_i(s)\,\textrm{d}s \end{aligned}$$

This formulation accounts for the projected history of the longitudinal outcome (i.e. true/latent MD) from a user-specified initial time (age), $t_0$, up to the current time (age), *t*, in the predictor of the relative risk submodel.

In the Results section we illustrate, with an example, specifically for functions (5) and (6), how choice of function impacts the relationship between the marker trajectory and (instantaneous) risk.

### Specific model formulations

For the longitudinal submodel, we included an effect of MHT use (never used/former use/current use), BMI (continuous variable), and MP status (pre/post) as variables at baseline. We transformed MD by taking its square root, to have a normal distribution, prior to including it in the longitudinal submodel. To account for a nonlinear trend, we applied a natural cubic spline within the mixed-effects models (in both the fixed and random effects). We allowed the trend over time to differ according to MHT treatment and MP status. This, in an approximate manner, accounts for individual variation in the ages at which women enter menopause. We fitted the model:

7 $$\begin{aligned} y_i(t_{ij})&= (\beta _0 +b_{i0})+(\beta _1 +b_{i1}) B_n(t,\lambda _1)+(\beta _2 +b_{i2})B_n(t,\lambda _2)+(\beta _3 +b_{i3})B_n(t,\lambda _3) \\&\quad +\beta _4 B_n(t,\lambda _1) MHT_{1i} +\beta _5 B_n(t,\lambda _2) MHT_{1i}+\beta _6 B_n(t,\lambda _3) MHT_{1i} \\&\quad +\beta _7 B_n(t,\lambda _1) MHT_{2i} +\beta _8 B_n(t,\lambda _2) MHT_{2i}+\beta _9 B_n(t,\lambda _3) MHT_{2i} \\&\quad +\beta _{10} B_n(t,\lambda _1) MP_{1i} +\beta _{11} B_n(t,\lambda _2) MP_{1i}+\beta _{12} B_n(t,\lambda _3) MP_{1i} \\&\quad +\delta _1 BMI_i+\delta _2 MHT_{1i}+\delta _3 MHT_{2i}+ \delta _4 MP_{1i}+ \epsilon _{ij}, \ \epsilon _{ij} \sim N(0,\sigma ^2) \end{aligned}$$

where ${B_n(t,\lambda _k): k = 1,2,3}$ denotes the B-spline basis matrix for a natural cubic spline with knots $\lambda _k$: interior knots at ages 50 and 55 years were chosen, while boundary knots at ages 43 and 65 years constrained the trends to be linear beyond these ages. We note that the multivariate distribution of the random effects naturally handles that baseline MD will be strongly associated with the level of MD reduction across time.

For the time-to-event (cancer diagnosis) process, a relative risk model with a penalized-spline-approximated baseline risk function was used in all cases. We included BMI, MHT, and family history (FH) of BC (No/yes/missing) as covariates. We used a time scale based on age since 40, as 40 is the lower limit of the screening age interval. The majority of women entered the study over the age of 40, which means that they had delayed entry times. The resulting left truncation was accounted for in our analysis. The hazard is modelled as:

8 $$\begin{aligned} h_i(t)&= h_0(t)\ \text {exp}(\gamma _1 BMI_i+ \gamma _2 MHT_{1i}+\gamma _3 MHT_{2i} +\gamma _4 FH_{1i}+\gamma _5 FH_{2i}\\&\quad + f(m_i(t),b_i,\alpha )) \end{aligned}$$

### Software

We used the JMbayes package in R for our analysis. A study [36] reviewing seven available JM packages in R concluded that, JMbayes [26] is the most comprehensive and expandable one. JMbayes allows for flexibility in the modeling of parametric and nonparametric baseline hazards, spline-based nonlinear longitudinal trajectories, and different association structure parameterizations. It also handles left truncation.

### Model comparisons and Bayes *p* values

Because we use Bayesian inference software, in order to compare the different JMs in terms of goodness-of-fit, we rely on the Deviance Information Criterion (DIC), which is a hierarchical modeling generalization of the Akaike information criterion [37]. Smaller values indicate better model adjustments to the data. Reported point estimates of parameters are posterior means, and p-values are Bayes p-values, based on tail probabilities for containing the value zero [26].

## Results

Key characteristics of individuals included in our analyses are described in Table 1. The average length of follow-up was 5.44 years. The time interval between mammography rounds in this cohort was 18–24 months. The majority of women (76.3%) had completed 3 or more rounds of mammography. The maximum number of rounds of mammography was six. 518 women were diagnosed with BC during follow-up. Those women were older, more likely to be postmenopausal, to be using MHT, and to have a first-degree relative that has been diagnosed with BC, than women without a BC diagnosis by end of follow-up. Women who developed breast cancer were older at baseline and had fewer screens compared to the women who did not develop breast cancer during follow-up. This is a bi-product of the strong relationship between age and breast cancer risk. The women entering at younger ages have lower chance of being diagnosed during follow-up (and consequently have more screens) than those entering at older ages.

**Fig. 1:**
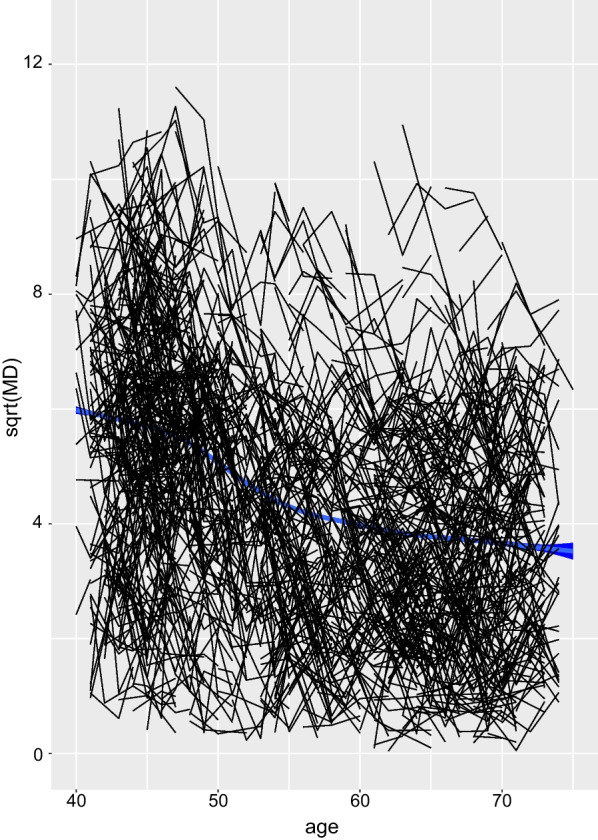
MD trajectory with age. Smoothed average (square root) MD values (all women), with age (the line is encapsulated within a 95% confidence band marked in blue), and individual trajectories of (square root) MD for a sample of 1000 women (black)

**Table 1:** Key characteristics at baseline of individuals included in the study

Characteristic	BC diagnosis by end of follow-up		All
	Yes	No	
Number	518	39,569	40,087
Invasive	443 (85.5%)	–	–
In-situ	75 (14.5%)	–	–
Screen detected	374 (72.2%)	–	–
Symptomatic	144 (27.8%)	–	–
No. of screens			
2 rounds	317 (61.2%)	9210 (23.3%)	9527 (23.7%)
3 rounds	176 (34.0%)	24,832 (62.7%)	25,008 (62.4%)
4 rounds	25 (4.8%)	5418 (13.7%)	5443 (13.6%)
5 or 6 rounds	–	109 (0.3%)	109 (0.3%)
MHT use			
Never	387 (74.7%)	32,513 (82.2%)	32,900 (82.1%)
Past	96 (18.5%)	5608 (14.2%)	5704 (14.2%)
Current	35 (6.7%)	1448 (3.6%)	1483 (3.7%)
Age at baseline	57 (9.4)	53.5 (9.3)	53.5 (9.3)
BMI	25.5 (4.1)	25.1 (4.1)	25.2 (4.2)
sqrt(MD)	5 (2.3)	4.8 (2.3)	4.8 (2.3)
MP status			
Pre	191 (36.9%)	19,285 (47.7%)	19,476 (48.6%)
Post	327 (63.1%)	20,284 (51.3%)	20,611 (51.4%)
FH			
Yes	112 (21.6%)	5249 (13.3%)	5361 (13.4%)
No	392 (75.7%)	33,290 (84.1%)	33,682 (84.0 %)
Missing	14 (2.7%)	1030 (2.6%)	1044 (2.6%)

Means (with standard deviations) are given for continuous variables and proportions (with percentages) are given for categorical variables

**Table 2:** Parameter estimates and 95% credibility intervals for the event process under the joint modeling analysis with different association structures

	Coefficient	2.5%	97.5%	Exp(Coeff.)*	*P*
Model (1)					
BMI ($\gamma _1$)	0.034	0.009	0.055	1.034	0.003
MHT$_1$ ($\gamma _2$)	− 0.078	− 0.306	0.156	0.925	0.507
MHT$_2$ ($\gamma _3$)	0.400	0.016	0.728	1.492	0.039
FH$_1$ ($\gamma _4$)	0.518	0.280	0.717	1.678	0.000
FH$_2$ ($\gamma _5$)	0.060	− 0.476	0.571	1.062	0.790
Current value ($\alpha$)	0.130	0.081	0.174	1.139	0.000
Model (2)					
BMI ($\gamma _1$)	0.034	0.008	0.057	1.035	0.007
MHT$_1$ ($\gamma _2$)	− 0.087	− 0.322	0.153	0.917	0.509
MHT$_2$ ($\gamma _3$)	0.400	0.020	0.730	1.492	0.037
FH$_1$ ($\gamma _4$)	0.514	0.305	0.718	1.672	0.000
FH$_2$ ($\gamma _5$)	0.058	− 0.487	0.538	1.060	0.777
Current value ($\alpha _1$)	0.120	0.071	0.168	1.127	0.000
Current slope ($\alpha _2$)	− 1.160	− 2.003	− 0.334	0.313	0.005
Model (3)					
BMI ($\gamma _1$)	0.039	0.019	0.060	1.040	0.000
MHT$_1$ ($\gamma _2$)	− 0.067	− 0.296	0.170	0.964	0.612
MHT$_2$ ($\gamma _3$)	0.404	0.019	0.725	1.498	0.036
FH$_1$ ($\gamma _4$)	0.511	0.301	0.720	1.667	0.000
FH$_2$ ($\gamma _5$)	0.064	− 0.506	0.572	1.066	0.788
Cumulative value $(\alpha$)	0.008	0.005	0.011	1.008	0.000

BMI: body mass index; MHT: menopausal hormone treatment (MHT_1_=former use, MHT_2_=current use); FH: family history (FH_1_=yes, FH_2_=missing); *: represents multiplicative change in risk per 1-unit increase in the parameter value

Figure 1 represents (the square root of) MD measurements (joined by lines) for 1000 randomly selected individuals. We smoothed the data points (for all KARMA women) using a natural cubic spline and added this (with 95% confidence band in blue) to the figure. Although this function represents an average, it has a pattern which is thought to be typical of an individual’s MD trajectory. MD is inversely and nonlinearly associated with age, with the largest declines observed between the ages of 45 and 60 years (during the menopausal transition) [9, 10, 13]. The large measurement error that contributes to the fluctuations in measured MD across time, within individuals, provides motivation for using a JM approach, i.e. to improve statistical efficiency.

Point estimates, with 95% credibility intervals, for all joint models—for parameters in the event and the longitudinal processes are shown in Tables 2 and 3, respectively. We refer to models using the time-to-event sub-models in Eqs. (4)–(6), as models (1)–(3), respectively. All models showed strong evidence of an association between MD trajectory and BC risk ($P_\alpha < 0.001$ for current value of MD in model (1), $P_{\alpha _1} < 0.001$ and $P_{\alpha _2} = 0.005$ for current value and slope of MD respectively in model (2), and $P_\alpha < 0.001$ for the cumulative value of MD in model (3)). According to the DIC criterion, the best model fit was obtained using the current value and slope of MD association structure (model (2), DIC = 1,074,546), followed by the cumulative association structure (model (3), DIC = 1,075,533), and then the current value of MD alone (model (1), DIC = 1,075,645). Model (1) suggested that a 1-unit increase in the value of the square root of MD corresponds to a exp(0.130) = 1.139-fold increase in the risk of BC diagnosis (2.5–97.5% credibility interval (CI): 1.084–1.190). Using model (2), we estimated that a 1-unit increase in the value of the slope is associated with a exp(− 1.160) = 0.313-fold decrease in the risk of BC diagnosis (2.5–97.5% CI 0.135–0.716)). This is counter-intuitive as it implies that reducing density increases risk. An explanation of this observation is given in the Discussion. Model (3) suggests that a unit increase in the area under the profile of the square root of MD corresponds to a exp(0.008) = 1.008-fold increase in risk (2.5–97.5% CI 1.005–1.011); Table 2.

**Table 3:** Parameter estimates and 95% credibility intervals for longitudinal submodels under the joint modeling analysis (2)

	Model(1)				Model (2)				Model (3)			
	Value	2.5%	97.5%	*P*	Value	2.5%	97.5%	*P*	Value	2.5%	97.5%	*P*
Intercept ($\beta _0$)	10.321	10.215	10.423	0.000	10.316	10.211	10.419	0.000	10.318	10.216	10.421	0.000
Spline$_1$ ($\beta _1$)	− 2.163	− 2.220	− 2.101	0.000	− 2.160	− 2.218	− 2.106	0.000	− 2.161	− 2.218	− 2.105	0.000
Spline$_2$ ($\beta _2$)	− 2.685	− 2.748	− 2.616	0.000	− 2.682	− 2.748	− 2.618	0.000	− 2.685	− 2.750	− 2.617	0.000
Spline$_3$ ($\beta _3$)	− 2.424	− 2.473	− 2.373	0.000	− 2.422	− 2.470	− 2.374	0.000	− 2.423	− 2.471	− 2.373	0.000
MHT$_1$ $\times$ Spline$_1$ ($\beta _4$)	0.302	0.183	0.419	0.000	0.301	0.189	0.412	0.000	0.293	0.181	0.410	0.000
MHT$_1$ $\times$ Spline$_2$ ($\beta _5$)	0.088	− 0.050	0.224	0.193	0.085	− 0.050	0.216	0.211	0.076	− 0.057	0.210	0.266
MHT$_1$ $\times$ Spline$_3$ ($\beta _6$)	0.229	0.124	0.332	0.000	0.231	0.129	0.330	0.000	0.223	0.121	0.328	0.000
MHT$_2$ $\times$ Spline$_1$ ($\beta _7$)	0.643	0.449	0.829	0.000	0.642	0.449	0.842	0.000	0.654	0.451	0.855	0.000
MHT$_2$ $\times$ Spline$_2$ ($\beta _8$)	0.253	0.024	0.479	0.033	0.252	0.023	0.483	0.029	0.265	0.027	0.492	0.025
MHT$_2$ $\times$ Spline$_3$ ($\beta _9$)	0.514	0.339	0.683	0.000	0.514	0.340	0.693	0.000	0.526	0.350	0.705	0.000
MP$_1$ $\times$ Spline$_1$ ($\beta _{10}$)	0.916	0.836	1.002	0.000	0.918	0.837	0.995	0.000	0.916	0.839	0.993	0.000
MP$_1$ $\times$ Spline$_2$ ($\beta _{11}$)	0.854	0.759	0.956	0.000	0.853	0.760	0.947	0.000	0.853	0.758	0.948	0.000
MP$_1$ $\times$ Spline$_3$ ($\beta _{12}$)	0.980	0.907	1.054	0.000	0.981	0.912	1.051	0.000	0.979	0.910	1.047	0.000
BMI ($\delta _1$)	− 0.179	− 0.183	− 0.175	0.000	− 0.179	− 0.183	− 0.175	0.000	− 0.179	− 0.183	− 0.175	0.000
MHT$_1$ ($\delta _2$)	− 0.035	− 0.116	0.042	0.395	− 0.036	− 0.117	0.047	0.388	− 0.029	− 0.117	0.053	0.505
MHT$_2$ ($\delta _3$)	0.174	0.038	0.318	0.012	0.177	0.040	0.310	0.013	0.167	0.029	0.306	0.013
MP$_1$ ($\delta _4$)	− 0.508	− 0.560	− 0.457	0.000	− 0.510	− 0.561	− 0.457	0.000	− 0.509	− 0.558	− 0.457	0.000
$\sigma$	0.547	0.539	0.554	0.000	0.546	0.539	0.554	0.000	0.547	0.539	0.554	0.000
D[1, 1]	3.873	3.474	4.249	0.000	3.896	3.500	4.281	0.000	3.874	3.475	4.258	0.000
D[2, 1]	− 3.435	− 3.788	− 3.096	0.000	− 3.432	− 3.782	− 3.093	0.000	− 3.374	− 3.725	− 3.018	0.000
D[3, 1]	− 4.088	− 4.628	− 3.551	0.000	− 4.109	− 4.653	− 3.558	0.000	− 4.073	− 4.627	− 3.501	0.000
D[4, 1]	− 3.652	− 4.003	− 3.297	0.000	− 3.627	− 3.974	− 3.266	0.000	− 3.613	− 3.945	− 3.257	0.000
D[2, 2]	9.195	8.219	10.142	0.000	9.210	8.245	10.149	0.000	9.139	8.078	10.193	0.000
D[3, 2]	8.287	7.642	8.952	0.000	8.260	7.630	8.910	0.000	8.158	7.512	8.816	0.000
D[4, 2]	7.120	6.373	7.859	0.000	7.141	6.379	7.871	0.000	7.093	6.308	7.884	0.000
D[3, 3]	12.147	10.781	13.515	0.000	12.147	10.749	13.486	0.000	12.109	10.646	13.510	0.000
D[4, 3]	6.486	6.027	6.964	0.000	6.436	5.953	6.920	0.000	6.421	5.938	6.906	0.000
D[4, 4]	7.077	6.321	7.822	0.000	7.085	6.326	7.845	0.000	7.054	6.295	7.783	0.000

Longitudinal outcome is square root of MD. *D*[*i*, *j*] denote the *ij*-element of the covariance matrix for the random effects

*BMI* body mass index, *MHT* menopausal hormone treatment, *MP* menopausal status

Table 3 shows that the estimated coefficients for the longitudinal process were similar in all JMs. In accordance with previous literature [38, 39], MD was negatively associated with BMI and MP, and was positively associated with MHT. Statistical significance of the spline interaction terms suggest that the models captured (to some degree) the influence of the timing of the menopause and MHT on the MD trajectory. For all models, the risk of being diagnosed with BC, was positively associated with current use of MHT and having a family history of BC.

**Fig. 2:**
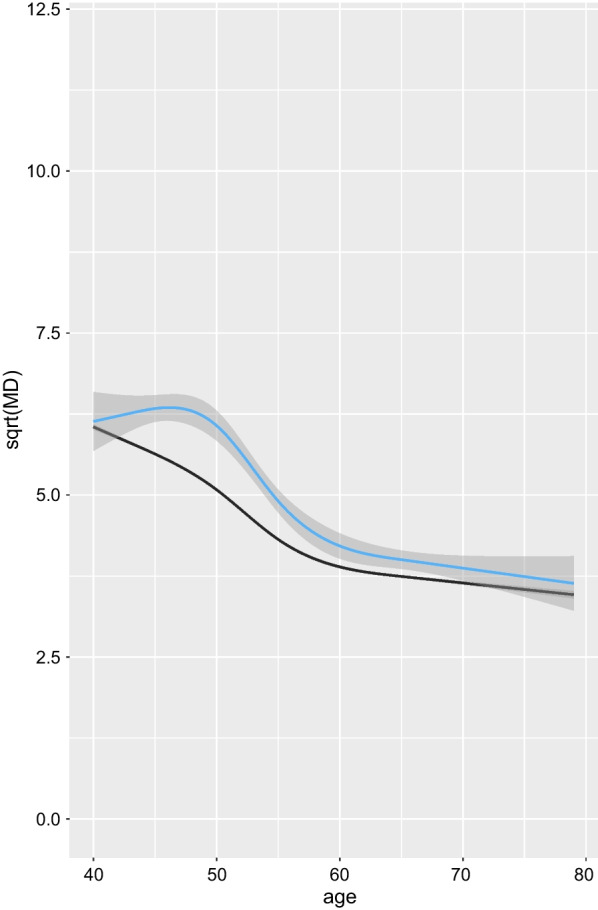
Smoothed average (square root) predicted MD values. Smoothed average (square root) predicted MD values (from model (3)), by age, for women diagnosed with BC at the end of follow-up (with 95% confidence band marked by shading in blue), and for women that remained free from BC diagnosis until the end of follow-up (in black—the confidence band is not represented since it is very narrow, comparable to that based on observed MD values for all women, represented in Fig. 1)

**Fig. 3:**
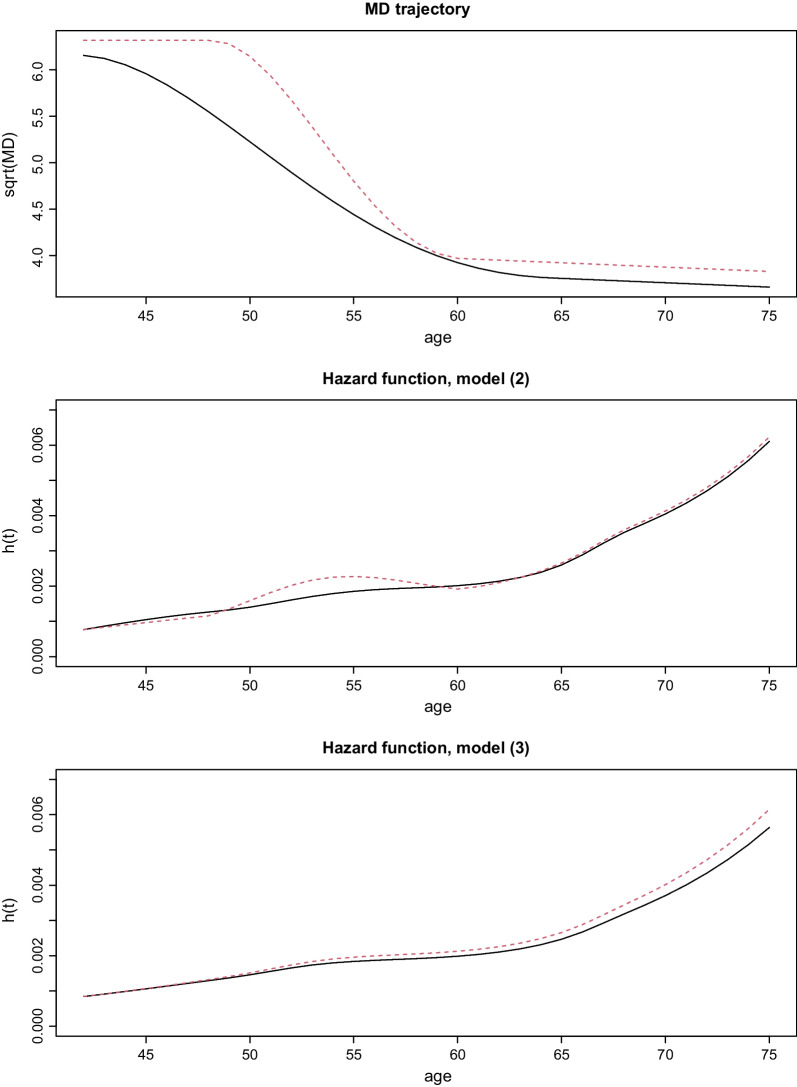
Predicted hazard functions for the two hypothetical individuals. Trajectories of (square root) MD of two hypothetical individuals in the upper panel. Predicted hazard functions for the two individuals, based on fitted models (2) and (3), are displayed in the middle and lower panel, respectively

For each individual we obtained predicted (square root) MD values across their follow-up period based on the fitted joint model (3). We smoothed these values using splines—separately for individuals with/without a BC diagnosis during follow-up; see Fig. 2. At young ages, on average, the women with a BC diagnosis appear to have slightly higher levels of density, and their density declines later on, than is the case for women without a BC diagnosis. For the other JMs, the corresponding plots were almost identical (data not shown). We also note that when we smooth the observed MD values separately for women with and without a BC diagnosis during follow-up, we see exactly the same pattern (data not shown).

To illustrate the significance of the estimates of the parameters of the time-to-event model, we estimated hazard functions for two hypothetical individuals with trajectories of (square root) of MD; Fig. 3. These were chosen to resemble the smoothed function in Fig. 2. Both individuals were assumed to have BMI values of 23 and to not have been using HRT. We note that for model (2) because of the negative coefficient for MD slope, the corresponding plots of the hazard functions crossed each other (see Discussion for an explanation).

## Discussion

Using data from a large prospective mammography screening cohort, we fitted three JMs with different association structures to investigate the association between MD and BC risk. The goodness-of-fit of both models that used (additional) characteristics of the MD trajectory, improved in comparison to using only the current value of MD.

Using only one measurement of MD, it has been consistently shown that women with a high MD have a substantially higher risk of BC than women with low MD; in [1], for example, women with 75% or greater MD were estimated to have an approximately 5-fold higher risk of BC compared to women with less than 10% dense tissue. In [28] which used a (Bayesian) JM with the current value of MD considered as a categorical variable using BIRADs, the mean of the posterior distribution of the hazard ratio was around four for category *d* (extremely dense) versus category *a* (fatty). Our results using model (1) yielded a similar effect size to these studies; women with current value of MD $>80$ cm$^2$ have $> 4$ fold higher risk of BC diagnosis than women with less than 25 cm$^2$. Studies based on analysing longitudinal data on MD, have focused on specific hypotheses—namely, current change of MD having a direct effect on current BC diagnosis [21, 22]. These studies have provided conflicting conclusions and have treated density change as a categorical variable (without accounting for measurement error).

The cumulative association structure-based model (model (3)) can be motivated by Boyd et al. [17] who argue that cumulative exposure to MD may reflect cumulative exposure to hormones that stimulate cell division in the breast and hence may be an important determinant of BC incidence. Our analysis is the first to examine this hypothesis by modelling individual level information, i.e. longitudinal data.

The observation obtained from model (2), that a high (instantaneous) decrease in MD is associated with an increased rate/risk of BC (diagnosis) is contrary to a hypothesis put forward in the literature, that women who do not experience a density decrease over time have a higher risk of BC than women who experience a decrease in MD [21–23]. The most likely explanation of the negative coefficient for density change in model (2) is that an instantaneous decrease in density can aid tumor detection, i.e. increase screening sensitivity. Some studies have specifically demonstrated how the age-related decrease in mammographic density is related to the increase of sensitivity of mammography with age [40, 41]. An alternative explanation could be related to the fact that breast tissue undergoes a massive remodeling during menopausal transition, known as lobular involution [42]. It has been shown that women with later ages at menopause have on average shorter menopausal transition periods than women with early ages at menopause. This might indicate a higher rate of change in women with late age at menopause, which might reflect a higher risk of BC [43]; see Fig. 2. We believe that the first explanation, that MD reduction increases sensitivity, is most likely, since we model diagnosis rather than the (unobservable) onset of cancer.

Because screening is not specifically incorporated in the modelling process, the effect of density change is, inappropriately, applied across all time points and can account e.g. for the unexpected crossing of hazard curves as observed in model (2) in Fig. 3 (in practice risk of diagnosis increases at the specific times that women attend screening and may particularly increase at these time points if density has decreased). For this reason, we suggest that the cumulative association structure JM is more robust (will give parameter estimates that more closely resemble biology) than the current MD value, current MD slope structure.

Clearly there is a lag between tumor onset and detection (even more problematic is that the duration of this lag varies dramatically across individuals). A pragmatic approach to account for this could be to model lagged effects of MD characteristics on diagnosis. JMs allow for lagged effects parameterizations [26]. In the current study we did not fit such models since almost 50% of the cases did not have images more than 2.5 years before diagnosis.

Because of the nature of the study design cases had fewer screening rounds than controls (follow-up stops at diagnosis). In theory, however, a bias could be introduced if, in the general population, the disease process is associated with the visit/screening process. There are JM approaches for additionally modeling the visiting process [44], but it has been shown [45] that even when the disease process is associated with the visiting process, fitting random effects models ignoring the visiting process produces estimates with little bias.

We should note that the benefits of JM are strictly linked to the correct specification of the longitudinal marker trajectory and the baseline hazard function, indicating the need for a careful consideration of assumptions to avoid biased estimates [46]. Although there are limitations to JMs for studying the MD–BC association, JMs do provide a better framework than approaches previously used in the literature - in particular because they account for measurement error. Using longitudinal trajectories of MD that account for measurement error can be less influenced by mammography acquisition parameters, such as compressed breast thickness.

A limitation of the current study is that we lacked longitudinal information on established BC risk factors. All information on covariates was collected at baseline, or at date of the first mammogram. A recent study advocated the need for longitudinal studies assessing the impact of breast fat and body weight history on MD features and BC risk over a long follow-up among both pre-menopausal and postmenopausal women [47]. Multivariate joint modeling can offer a useful tool to determine the interrelationships between BC risk factors and MD, and their relationship with risk of BC. In addition, integrating information about MD in younger women using different imaging techniques, such as MRI [17], would be highly valuable to increase our understanding of breast density through a woman’s lifetime.

In our study we used JMs that assume that parameters measuring the strength of the association between MD and BC diagnosis are time-constant. However, it might be more natural and more, biologically, relevant to assume that the effects of characteristics of the MD trajectory changes over time. Further work is needed based on JMs with time-varying association structures [48].

## Conclusion

Our study is the first to have used JMs to investigate the association between the longitudinal history of MD and BC risk whilst considering different association structures. We statistically investigated the hypothesis that the cumulative exposure to MD is associated with BC risk, which has been proposed earlier with biological arguments. A JM suggested that a decrease in MD may be associated with an increased (instantaneous) BC risk. It is possible that this is because of increased sensitivity rather than being related to biology. For this reason, the cumulative exposure association structure may represent a more useful model to study BC diagnosis.

## Data Availability

The datasets used and/or analysed during the current study are available from the corresponding author upon reasonable request and with permission of www.karmastudy.org.

## Abbreviations

MD: Mammographic density
BC: Breast cancer
JM: Joint model
BMI: Body mass index
MHT: Menopausal hormone treatment
MP: Menopausal status
FH: Family history
BIRADS: Breast Imaging Reporting and Data System
